# A comparison of maternal and neonatal outcomes between water immersion during labor and conventional labor and delivery

**DOI:** 10.1186/1471-2393-14-160

**Published:** 2014-05-06

**Authors:** Yinglin Liu, Yukun Liu, Xiuzhi Huang, Chuying Du, Jing Peng, Peixian Huang, Jianping Zhang

**Affiliations:** 1 Department of Obstetrics and Gynecology, Sun Yat-sen Memorial Hospital of Sun Yat-sen University, No 107, Yanjiang West Road, Guangzhou, Guangdong 510120, China; 2 Key Laboratory of malignant tumor gene regulation and target therapy of Guangdong Higher Education Institutes, Sun Yat-sen University, Guangzhou, Guangdong, China; 3 Department of Obstetrics and Gynecology, HuiZhou Municipal Central Hospital, Huizhou, PR, China; 4 Department of Obstetrics and Gynecology, GuangDong Women and Children Hospital, Guangzhou, PR, China

**Keywords:** Water birth, Water immersion during labor, Analgesia, Labor duration, Puerperal infection

## Abstract

**Background:**

Water immersion during the first stage of labor can reduce the length of the first stage and epidural/spinal analgesia use; however, there is limited information regarding other outcomes. Our purpose was to compare maternal and neonatal outcomes of women who underwent water immersion during the first stage of labor with those who underwent conventional labor and delivery.

**Methods:**

Healthy primipara with singleton pregnancies and cephalic presentation were included in the study. Patients were allowed to choose water immersion during labor or conventional labor and delivery. For water immersion, the water temperature was maintained at 35-38°C and subjects left the tub on complete cervical dilatation. A visual analogue scale (VAS) was used to assess pain during labor. Other outcome measures included duration of labor, type of delivery, blood loss, pelvic floor dysfunction and symptoms of stress urinary incontinence (SUI) at 42 days after delivery, infant Apgar scores, and need for transfer of the infant to the neonatal intensive care unit.

**Results:**

Thirty eight subjects (mean age, 28.66 ± 3.08 y) received water immersion and 70 (mean age, 27.89 ± 2.99 y) underwent conventional labor and delivery. There were no differences in maternal height, weight, age, gestational age, gravidity, and newborn weight between the groups (all, p>0.05). VAS pain scores were significantly greater in the conventional labor group at 30 min and 60 min after a cervical dilatation of 3 cm (30 min: 10 [9, 10] vs. 6 [5, 8]; 60 min: 10 [10, 10] vs. 7 [6, 8], respectively, both, p<0.001). The duration of labor and postpartum bleeding were similar between the groups (all, p>0.05). The cesarean section rate was higher in the conventional labor group (32.9% vs. 13.2%, p=0.026). The 1-minute and 5-minute Apgar scores were similar between the groups. Maternal and neonatal culture results were similar between the groups. SUI symptoms at 42 days after delivery was significantly higher in the conventional labor group (25.5% vs. 6.1%, respectively, p=0.035).

**Conclusions:**

Water immersion can reduce labor pain, and is associated with a lower rate of cesarean delivery and SUI symptoms at 42 days.

## Background

In a water birth, the woman labors and delivers in a tub of warm water. Studies have shown that water birth is associated with shorter labor, less use of analgesics, and less severe vaginal and perineal lacerations [1-6]. Warm water reduces the release of catecholamines in the body, increases uterine perfusion, enhances uterine rhythmic contractions, accelerates cervical dilation, and shortens the duration of labor [7,8]. Warm water can also increase the flexibility of the birth canal and perineum, facilitate the extension of the perineum and the birth canal, and reduce the pain of uterine contractions [1,7,9]. The disadvantages of water birth are that the perineum cannot be protected and episiotomy cannot be performed, the risk of perineal laceration is high, and continuous fetal heart rate monitoring cannot be performed in water. The newborn may also inhale contaminated water, which may increase the risk of neonatal aspiration syndrome and neonatal asphyxia [10].

Water immersion during the labor refers to immersion in water during only the first stage of labor; the delivery is not performed in the water. Water immersion may provide the advantages described above without the potential risks associated with water birth. This purpose of the study was to compare the maternal and neonatal outcomes of women who underwent water immersion during the first stage of labor with those who underwent conventional labor and delivery.

## Methods

### Subjects

Healthy primipara with singleton pregnancies and cephalic presentation who were hospitalized at Obstetrical Department of Sun Yat-sen Memorial Hospital, Sun Yat-sen University for delivery from June 2009 to February 2011 were included in the study. The hospital is a tertiary care hospital that performs approximately 2,000 deliveries per year. At our hospital, water immersion is an option for all patients, However, because of traditional Chinese believe that mother should avoid water and based on our rather restrict inclusion and exclusion criteria listed below, less than 100 cases choose water immersion service. Patients were allowed to choose water immersion during labor or conventional labor and delivery. Inclusion criteria were 1) regular prenatal examinations at the outpatient clinic; 2) between 20 to 35 years of age; 3) gestation age ≥ 37 weeks and < 42 weeks; 4) estimated fetal weight ≥ 2,500 g and < 3,500 g; and 5) no contraindications to vaginal delivery. Exclusion criteria were 1) pelvis stenosis; 2) complications of pregnancy making vaginal delivery contraindicated; 3) infectious diseases including hepatitis B, hepatitis C, syphilis, and HIV infection); and 4) untreated vaginal infection. This study was approved by the Institutional Review Board of the hospital and all patients provided written informed consent.

### Water immersion during labor

A vaginal examination was performed to 1) assess cervical dilation, cervical hardness, and cervical edema; 2) determine fetal position, fetal presentation, and whether overlapping cranial sutures were present; 3) to determine if rupture of membranes had occurred and 4) to perform internal pelvic measurements to assess for the possibility of cephalopelvic disproportion. If a subject chose water immersion and there were no contraindications, a soapy water enema was administered and the patient showered before the cervix was dilated to 3 cm. After the cervix dilated to 3 cm, the subject entered the warm water tube. During water immersion the subject was accompanied by her spouse or significant other and the obstetrician or midwife, and she was encouraged to drink water. Attention was paid to symptoms of dehydration (including rapid heart rate, hidrosis, dizziness, and nausea and vomiting) to avoid circulatory failure. The water temperature was maintained at 35-38°C. The subject was encouraged to leave the tub and rest for 30 minutes after every 2 hours of water immersion. Patients were not allowed to enter the water until they were dilated 3 cm in order to reduce the time in the warm water. Bathing in warm water results in dilation of blood vessels on the body surface, which can potentially result in tachycardia and hypotension, as well as dehydration. Patients were required to leave the tub intermittently to minimize the possibility of this. The patient was removed from the tub if she experienced abnormal discomfort, felt too hot or cold, if their heart rate or blood pressure were abnormal, or if the fetal heart rate tracing was abnormal.

The fetal heart rate was measured once every 15 min during water immersion, and maternal blood pressure, pulse, respiration, and blood oxygen were measured once every 30 minutes. Fetal heart rate was monitored in one of 2 ways. The first is the use of a waterproof Doppler probe. Second, the water immersion tub has different depth levels and thus the abdomen can be exposed out of the water and a standard fetal heart rate monitor can be used. At our hospital, a waterproof Doppler probe is more commonly used. For determination of blood pressure, the woman’s arm can be rested on the edge of the tub and blood pressure can be measured with a standard electronic sphygmomanometer. This also applies to measuring oxygen saturation. Vaginal examinations were performed when deemed necessary by the obstetrician or midwife. After full cervical dilatation the subject left the tub and was placed on a normal delivery bed.

### Outcome measures

A visual analogue scale (VAS) was used to assess pain during labor. Pain was scored on a scale of 0 to 10 with 0 = no pain and 10 = worst pain imaginable. Pain scores were assessed when the cervical dilatation was 3 cm before entering the tub, and 30 and 60 minutes after entering the tub. Pain scores were measured at 30 and 60 minutes after entering the tub for 2 reasons. 1) It takes about 30 minutes for the analgesia effect of the warm water to occur. 2) As suggested by the results of a prior study, the analgesic effect of the water immersion is stable after 30 minutes, and the effect didn’t change dramatically after immersion, therefore a 30-minute interval (60 minutes in the tub) was chosen for statistics analysis.

Culture specimens were obtained from the posterior fornix of the vagina 24 hours after delivery for subjects in both groups that delivered vaginally. Culture specimens from all neonates were collected from the posterior pharyngeal wall. Specimens were immediately placed in a sterile culture tube and sent for routine bacterial culture and identification. If no colony growth was observed at 48 hours, the culture was considered negative. Other outcome measures included duration of labor, type of delivery, blood loss, pelvic floor dysfunction and symptoms of stress urinary incontinence (SUI) at 42 days after delivery, infant Apgar scores and need for transfer of the infant to the neonatal intensive care unit. After childbirth, the subjects who chose water immersion completed a survey on maternal satisfaction, and they were asked whether they would choose water immersion during labor again for their next delivery.

### Statistical analysis

Continuous data were summarized as mean ± standard deviations (SD) for normally distributed data and median (interquartile range [IQR]: 1st and 3rd quartiles) for non-normally distributed data. Categorical data were summarized as number (percentage). Differences between groups were compared using two-sample t-test for continuous data with a normal distribution and Mann–Whitney U test for non-normally distributed data. Pearson Chi-square test or Fisher’s exact test, as appropriate, was used to compare categorical data. All statistical assessments were two-tailed, and a value of p < 0.05 was considered to indicate statistical significance. Statistical analyses were performed using SPSS 17.0 statistics software (SPSS Inc., Chicago, IL, USA).

## Results

A total of 108 subjects who met the inclusion criteria were enrolled in this study; 38 received water immersion during labor and 70 underwent conventional labor and delivery (Table 1). The mean maternal age in the water immersion group was 28.66 ± 3.08 y and in the conventional labor group was 27.89 ± 2.99 y (p > 0.05). There were no differences in maternal height, weight, gestational age, gravidity, and newborn weight between the groups (all, p > 0.05). The cesarean section rate was significantly higher in the conventional labor group (32.9% vs. 13.2%, respectively, p = 0.026) (Table 1). The indications for cesarean delivery in the water immersion group were fetal distress (n = 3), persistent occiput transverse position (n = 1), and prolonged second stage of labor (n = 1), and the indications in the conventional labor group were requested caesarean section for pain or social reasons (n = 8), fetal distress (n = 6), prolonged second stage (n = 5), and persistent occiput transverse position (n = 4) (data on file). The 1-minute and 5-minute Apgar scores were similar between the 2 groups (p = 0.333 and 0.231, respectively). Four (10.5%) infants in the water immersion group and 11 (15.7%) required transfer to the pediatrics unit after delivery (p = 0.568).

**Table 1:** Subject demographic and clinical characteristics and neonatal outcomes

**Variables**	**Water Immersion (n = 38)**	**Conventional Labor (n = 70)**	**p-value**
Age, y	28.66 ± 3.08	27.89 ± 2.99	0.207
Height, cm	159.66 ± 4.41	160.24 ± 4.4	0.513
Weight, kg	66.09 ± 7.48	65.17 ± 7.77	0.553
Gestational age, wk	39.73 ± 1.02	39.59 ± 1.00	0.492
Gravidity	1.39 ± 0.64	1.57 ± 0.81	0.247
Delivery method			0.026^*^
Vaginal	33 (86.8)	47 (67.1)	
Cesarean	5 (13.2)	23 (32.9)	
Transfer to pediatrics unit			0.568
Yes	4 (10.5)	11 (15.7)	
No	34(89.5)	59 (84.3)	
Newborn weight, g	3266.71 ± 317.52	3230.71 ± 356.53	0.604
Newborn Apgar score			
1 minute	10 (9 , 10)	10 (9 , 10)	0.333
5 minute	10 (10 , 10)	10 (10 , 10)	0.231

Continuous data were summarized as mean ± standard deviation (SD) for normally distributed data and median (interquartile range [IQR] for non-normally distributed data; categorical data were summarized as number (percentage).

*p < 0.05, indicates statistically significant difference between groups.

Intra- and postpartum data of the 80 subjects that delivered vaginally (33 in water immersion group and 47 in the traditional group) are shown in Table 2. VAS pain scores were significantly greater in the conventional labor group at both 30 min and 60 min after a cervical dilatation of 3 cm (30 min: 10 [9, 10] vs. 6 [5, 8]; 60 min: 10 [10] vs. 7 [6, 8], respectively, both, p < 0.001). The duration of each stage of labor was similar between the 2 group, as was the amount of intra- and postpartum bleeding (all, p > 0.05). The rate of SUI symptoms at 42 days after delivery was significantly higher in the conventional labor group than in the water immersion group (25.5% vs. 6.1%, respectively, p = 0.035).

**Table 2:** Intra- and postpartum outcomes of 80 subjects that had vaginal deliveries

**Variables**	**Water Immersion (n = 33)**	**Conventional Labor (n = 47)**	**p-value**
**Duration of labor, min**			
First stage	596.55 ± 249.71	552.30 ± 241.85	0.429
Second stage	58.79 ± 31.37	56.04 ± 35.15	0.720
Third stage	10.88 ± 6.14	9.94 ± 5.24	0.463
Total	666.55 ± 259.70	618.32 ± 252.30	0.408
**Bleeding, mL**			
Intrapartum	188.79 ± 107.03	205.32 ± 152.37	0.593
24 h postpartum	324.39 ± 125.84	367.02 ± 239.72	0.353
**VAS pain score**			
Cervical dilation of 3 cm^a^	10 (7 , 10)	10 (8 , 10)	0.776
30 min after cervical dilation of 3 cm^b^	6 (5 , 8)	10 (9 , 10)	<.001^*^
60 min after cervical dilation of 3 cm^c,d^	7 (6 , 8)	10 (10, 10)	<.001^*^
**Pelvic floor dysfunction**			
SUI symptoms during pregnancy	6 (18.2)	15 (31.9)	0.169
SUI symptoms 42 days postpartum	2 (6.1)	12 (25.5)	0.035^*^
POP 42 days postpartum	8 (24.2)	12 (25.5)	0.896
**Pelvic floor muscle strength 42 days postpartum**			0.766
0	3 (9.1)	4 (8.5)	
1	6 (18.2)	8 (17.0)	
2	11 (33.3)	15 (31.9)	
3	12 (36.4)	18 (38.3)	
4	1 (3.0)	2 (4.3)	

VAS, visual analogue scale; SUI, stress urinary incontinence; POP, pelvic organ prolapse.

Continuous data were summarized as mean ± standard deviation (SD) for normally distributed data and median (interquartile range [IQR]) for non-normally distributed data; categorical data were summarized as number (percentage).

^a^Experimental group, VAS evaluation when cervical dilation of 3 cm before entering the tub; conventional group, VAS evaluation when cervical dilation of 3 cm.

^b^Experimental group, VAS evaluation 30 min after cervical dilation of 3 cm and in the tub; conventional group, VAS evaluation 30 min after cervical dilation of 3 cm.

^c^Experimental group, VAS evaluation 60 min after cervical dilation of 3 cm and in the tub; conventional group, VAS evaluation 60 min after cervical dilation of 3 cm.

^d^Complete data were not available for 12 subjects (5 in water immersion group and 7 in conventional labor group) due to the subject leaving the water tank or completing delivery.

*p < 0.05, indicates statistically significant difference between groups.

Maternal vaginal and neonatal oral culture results are shown in Table 3. Maternal vaginal cultures were positive in 5 patients in the water immersion group and 7 in the conventional labor group. In the water immersion group, 2 cultures were positive for Gram-positive bacilli and one was positive for *Streptococcus agalactiae*, *Escherichia coli*/*Enterococcus faecalis*, and *fungi*, respectively. In the conventional labor group 3 cultures were positive for Gram-positive bacilli, 2 for fungi, and 1 for *Staphylococcus lugdunensis* and Group B *Streptococcus*, respectively. Neonatal oral swab culture results were positive in 1 infant in the water immersion group and 2 in the conventional labor group. The culture in the water immersion group was positive for *Escherichia coli*/*Enterococcus faecalis*, and the 2 in the conventional labor group were positive for Gram-positive bacilli.

**Table 3:** Maternal and neonatal bacterial culture results

	**Maternal vaginal swab 24 h after delivery**			**Neonatal oral swab**		
	**Water immersion (n = 33)**	**Conventional labor (n = 47)**	**p-value**	**Water immersion (n = 33)**	**Conventional labor (n = 47)**	**p-value**
**Total positive**	5	7	1.000	1	2	1.000
Gram-positive bacilli	2 (6.1)	3 (6.4)		0 (0)	2 (4.3)	
Streptococcus agalactiae	1 (3.0)	0 (0)		0 (0)	0 (0)	
Escherichia coli/Enterococcus faecalis	1 (3.0)	0 (0)		1 (3.0)	0 (0)	
Fungi	1 (3.0)	2 (4.3)		0 (0)	0 (0)	
Staphylococcus lugdunensis	0 (0)	1 (2.1)		0 (0)	0 (0)	
Group B Streptococcus	0 (0)	1 (2.1)		0 (0)	0 (0)	

Data were summarized as number (percentage).

After childbirth, the subjects who experienced water immersion completed a survey on maternal satisfaction; 2 of the 38 subjects were very satisfied and 36 were satisfied with the effect of water immersion during labor (data not shown).

## Discussion

The results of this study comparing water immersion during labor with conventional labor and delivery showed that water immersion during the labor can reduce the labor pain and is associated with a lower rate of cesarean delivery. Water immersion does not increase the rate of maternal or neonatal infections, but is associated with a lower rate of SUI symptoms at 42 days postpartum. At our hospital, all patients know about the water immersion service and based on the inclusion and exclusion criteria used in this study they are allowed to decide if they want to undergo water immersion.

### Analgesic effect of water immersion during labor

An ideal analgesic technique can significantly reduce pain during labor and childbirth, and have minimal impact on the fetus and the labor process, and there are both pharmacological and non-pharmacological methods available [8]. Regional anesthesia (epidural analgesia, spinal/epidural block, and continuous subarachnoid anesthesia) is the most effective labor analgesic method with the least sedative effects [8,11,12]. Of the regional anesthetic methods, epidural analgesia is safe and has little impact on the mother and child. Its administration relatively simply, its onset of action is rapid and the effects are reliable effects, the motor nerve block is mild, uterine contractions are not affected, and it allows cesarean delivery and assisted delivery when necessary. In 2006, American College of Obstetrics and Gynecology (ACOG) and the American Society of Anesthesiologists (ASA) reached a consensus that labor analgesia can be given as long as the patient has requested analgesia, and epidural anesthesia is the preferred method. However, the use of regional anesthesia and other pharmacological methods is affected by varying concepts of labor analgesia in different countries [13]. Although pharmacological methods of analgesia (regional anesthesia and systemic drugs such as meperidine) have a rapid onset of action and prominent analgesic effects, their side effects and complications cannot be ignored [12]. Non-pharmacological methods of labor analgesia include Doula support during labor and delivery, music therapy, postural changes, water immersion and birth, and acupuncture [8,12]. Non-pharmacological analgesic methods are simple to administer and have no side effects or adverse effects on the mother and child. They have gradually become popular; however, their efficacy still requires more comprehensive and in-depth research.

Cluett et al. [9] collected and analyzed the data of randomized controlled trials on water immersion during labor, and the results showed that water immersion during labor can alleviate labor pain and reduce the use of analgesics. Other studies have also indicated that immersion in water during labor and delivery can reduce the analgesic requirements of the mother without adverse effects on the neonate [2-4,14,15]. Our results also showed that VAS pain scores were significantly lower in the water immersion group than in the conventional labor group. Various theories have been postulated to explain why water immersion can reduce the pain of labor. The body's average specific gravity is less than that of water, thus the patients is in a relative state of weightlessness which allows the patient to assume various positions that are relaxing and comfortable. This, combined with the warmth of the water can produce a sedative effect which can alleviate stress and anxiety [7,8]. In addition, water immersion has been shown to decrease the secretion of catecholamines and other stress-related hormones [1]. Compared with regional analgesia, water immersion during the labor is convenient, comfortable, and has no side effects. No anesthesiologist is required, and there is no risk of trauma or the complications of anesthesia. Thus, water immersion during the labor is a relatively ideal method for providing analgesia during labor.

### Water immersion and birth outcomes

Unnecessary cesarean deliveries increase medical costs and as a major surgical procedure are associated with complications. Social factors such as searching for a faster and pain-less way to delivery due to the labor pain have been cited as one of the main reasons for the high rate of cesarean deliveries [16]. Since water immersion during the labor can alleviate labor pain and provide more personalized services for the mother during labor, it may reduce the number of cesarean deliveries performed for social factors, thereby reducing the cesarean section rate. Studies of water immersion during labor and water birth have shown that water immersion during labor is associated with a shorter labor as compared with conventional labor and delivery in a bed [4,5,15,17] as well as a lower cesarean delivery rate [15]. Cluett et al. [18] also reported water immersion may reduce the need for obstetrical interventions in women experiencing slow progress of labor (cervical dilatation < 1 cm/h in the active phase of labor). Though our results did not show a difference in the duration of labor between the 2 groups, the cesarean delivery rate in the water immersion group was significantly lower than that in the conventional labor group (13.2% vs. 32.9%, respectively, p = 0.026). Moreover, no cesarean deliveries in the water immersion group were performed for social factors. In our country, it is acceptable for a patient to request a cesarean delivery during labor. This is not a standard worldwide, but is part of our culture. In most cases patients request a cesarean section because of labor pain. The lower number of cesarean deliveries in the water immersion group supports the hypothesis that water immersion reduces labor pain as there were no cesarean deliveries performed for social reasons in the water immersion group.

Our results showed that neonatal outcomes were not different between the water immersion group and the conventional labor group. The potential for adverse neonatal outcomes has been an argument against water immersion and water births [19]. However, a number of studies have reported equivalent or better neonatal outcomes (Apgar scores, requirement for neonatal intensive care unit admission) for women that have undergone water immersion and water births as compared with those who have undergone conventional labor and delivery [2-4,20].

### Protective effect of water immersion during labor on pelvic floor function

Female pelvic floor dysfunction mainly includes pelvic organ prolapse (POP) and SUI, and there are no studies specifically examining the potential protective effects of water immersion during labor on pelvic floor function. Warm water immersion can reduce the pressure difference between the inside and outside vagina and improve the elasticity of the perineum, thereby reducing the possibility of damage to the birth canal [1,7]. Warm water can also increase the perineal blood circulation and may reduce damage caused by ischemia and hypoxia. Thus, theoretically water immersion during the labor can reduce pelvic floor tissue injury and subsequent pelvic floor dysfunction. Studies have shown that water immersion and water birth are associated with a lower use of episiotomy and less severe vaginal and perineal injuries than conventional labor and delivery [2-4,17]. The results of this study showed that the incidence of SUI symptoms at 42 days after childbirth in the water immersion group was significantly lower than that in the conventional labor group (6.1% vs. 25.5%, respectively, p = 0.035), suggesting that water immersion during the labor provides a protective effect on pelvic floor function at an early stage postpartum.

### Infections and water immersion

During water immersion, the water in the tub cannot be completely sterile and thus there is concern that water immersion during labor and water birth may increase the rate of maternal and neonatal infections. Thoeni et al. [5] reviewed 1,600 water births at a single institution over an 8 year period and reported that the neonatal infection rate of water births was similar to that of conventional labor and deliver (1.22% vs. 2.64%, respectively). Zanetti-Daellenbach et al. [21] compared the maternal and neonatal outcomes of women who received a water birth, water immersion and then conventional vaginal delivery, and labor and deliver without immersion and found that the maternal and neonatal infection rates were similar in the 3 groups. Cluett et al. [9] retrospectively studied 11 randomized controlled clinical trials and concluded that water immersion during the labor does not increase the rate of maternal and neonatal infections. The results of this study showed the positive rates of postpartum vaginal cultures and neonatal pharyngeal cultures were not different between the 2 groups. These results indicated that water immersion during the labor did not increase the risk of maternal and neonatal infections.

### Staff training for water immersion

Before beginning the service of water immersion during labor, trained labor and delivery staff at our hospital received 1 month of training at Urogynecology Center, C. H Saint-Philibert Lille and Clinique Adassa at Strasbourg in France. The staff obtained a certificate after passing the examination, and mastering the key techniques. Experts from the French hospital also came to our hospital for training sessions with all of the staff which included lectures, video presentations, and hands on training sessions. Also, staff with infectious diseases or with any type of skin lesion or dermatitis is excluded from caring for patients undergoing water immersion. In addition, those with physical conditions that prohibit them from assisting patients into and out of the tub, such as joint or spine diseases, do not care for patients undergoing water immersion.

There are a number of limitations to this study that should be considered. First, patients were allowed to choose water immersion or conventional labor, thus they were not randomized. Patients that choose water immersion may have had a bias towards believing that water immersion would be beneficial. However, the 2 groups were similar with respect to baseline demographic and clinical characteristics. In addition, the number of patients in the water immersion group was small. There are a number of reasons for this. 1) Water immersion is new and has not been widely promoted, and Chinese women are not familiar or trusting of it. The majority of mothers and their families still prefer conventional delivery methods. 2) The exclusion criteria are strict. For example, hepatitis B virus carriers are excluded, and this condition is common in the Chinese population. 3) The main advantage of water immersion before delivery is the analgesia effect, however, other methods of analgesia, such as Doula delivery, are available and patients are more familiar with these methods. A post hoc power analysis showed that the statistical power for detecting a difference in VAS pain score at both 30 min (traditional vs water immersion: 9.43 ± 0.91 vs. 6.29 ± 1.39) and 60 min (traditional vs. water immersion: 9.86 ± 0.40 vs. 7.03 ± 1.18) was 99.9%. Thus, although the sample size was small, the confidence is high that the difference was significant.

## Conclusions

In summary, the results of this study suggest that water immersion during the labor can reduce labor pain and is associated with a lower rate of cesarean delivery. Water immersion does not appear increase the rate of maternal or neonatal infections, but is associated with a lower rate of SUI symptoms at 42 days postpartum. Water immersion during the labor is an intrapartum service model that is worthy of promotion and application.

## Competing interests

The authors declare that they do not have any conflict of interest.

## Authors’ contributions

We declare that all the listed authors have participated actively in the study and all meet the requirements of the authorship. Yinglin Liu, Yukun Liu and Jianping Zhang designed the study and wrote the protocol, Yinglin Liu, Yukun Liu, Xiuzhi Huang and Peixian Huang performed research/study, Chuying Du and Jing Peng managed the literature searches and analyses, Xiuzhi Huang, Chuying Du and Peixian Huang undertook the statistical analysis, Yinglin Liu, Yukun Liu wrote the first draft of the manuscript. All authors read and approved the final manuscript.

## Pre-publication history

The pre-publication history for this paper can be accessed here:

http://www.biomedcentral.com/1471-2393/14/160/prepub
